# Supplementary material for: An expanded population of CD8dim T cells with features of mitochondrial dysfunction and senescence is associated with persistent HIV-associated Kaposi’s sarcoma under ART
Source: Front Cell Dev Biol. 2022 Sep 29;10:961021. doi: 10.3389/fcell.2022.961021 (PMC9557199; doi:10.3389/fcell.2022.961021)
Supplement: Supplementary file 1 [file DataSheet1.PDF]

Supplementary Materials

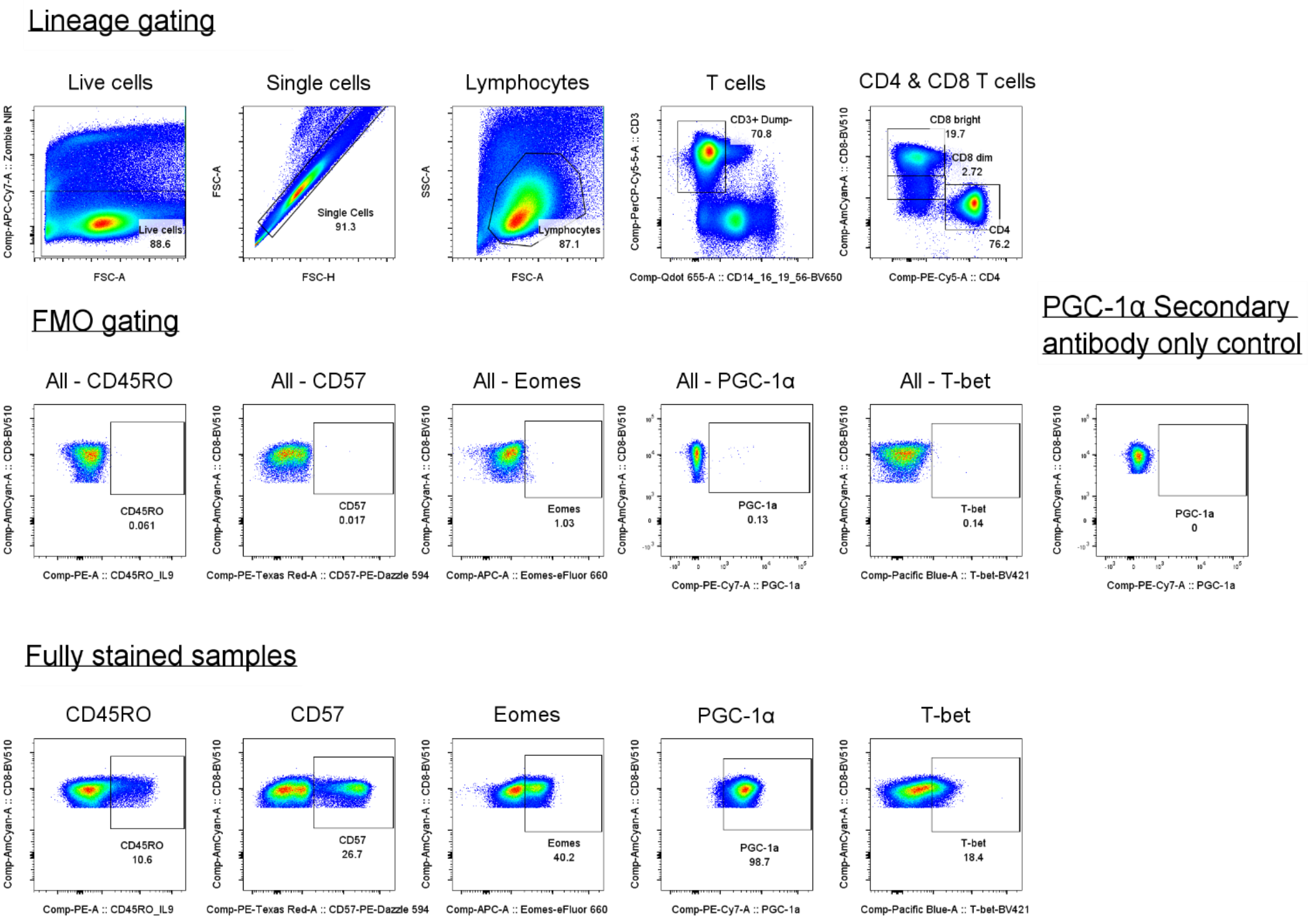

Supplementary Figure 1. Gating strategy used to identify CD8 bright and dim T cells (top row) and their expression of phenotypic markers (bottom row). Gates were set using fluorescence minus one (FMO) controls (middle row). For PGC-1α, the secondary antibody only control was used to gate positive events, as this staining produced slightly higher background than the FMO.

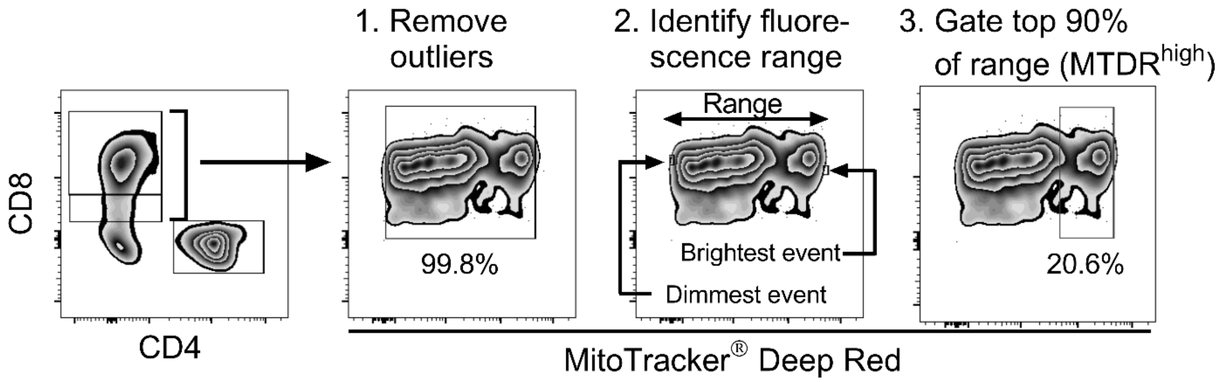

Supplementary Figure 2. Gating strategy used to delineate MitoTracker Deep Red (MTDR) "high" CD8 T cells. After excluding outliers (top and bottom 0.1%), the fluorescence intensity of the brightest and dimmest cells indicated the fluorescence range. Cells that fell within the top 90% of this fluorescence range were considered MTDR^{high}. (Note: plots use bi-exponential scale.)

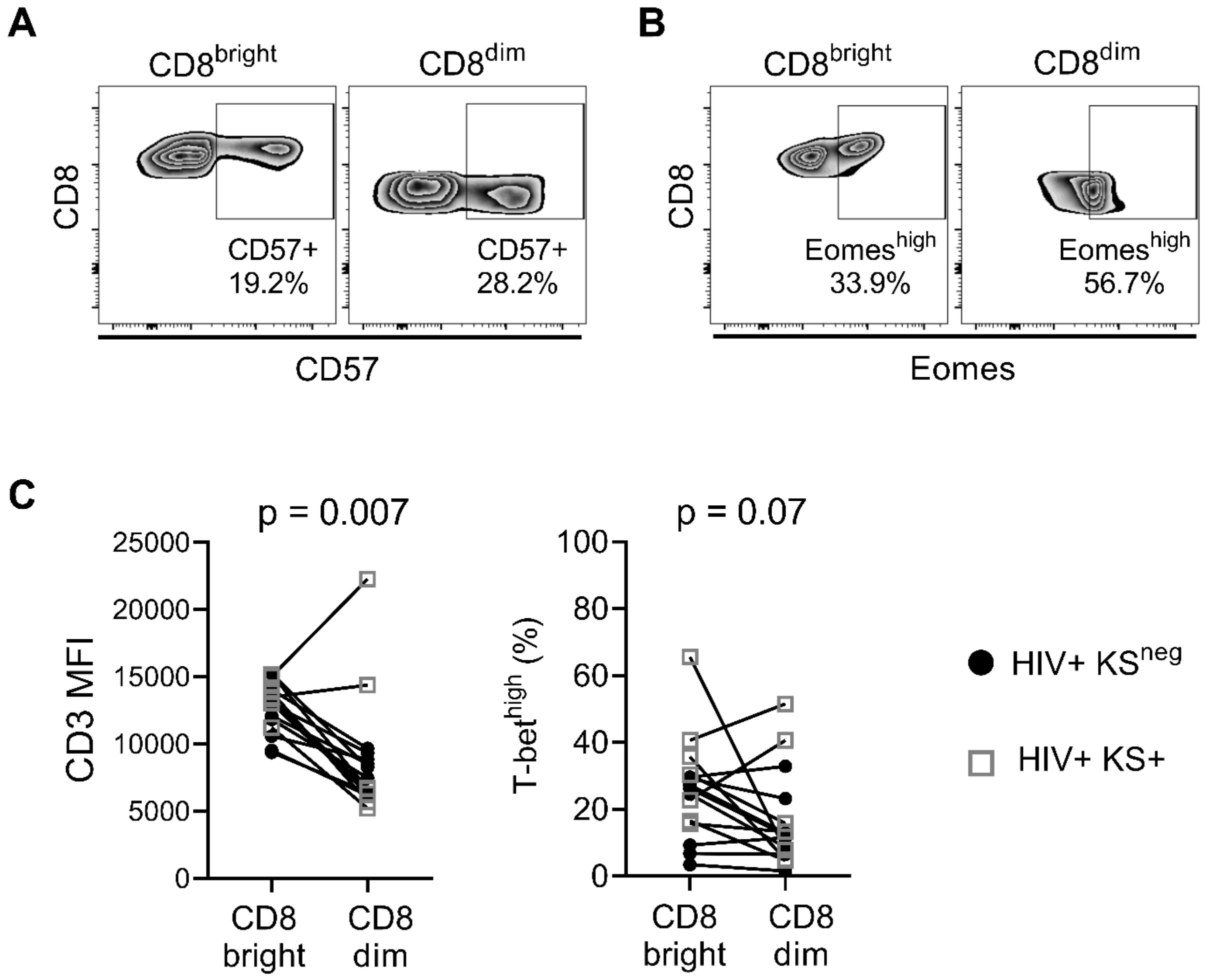

Supplementary Figure 3. A) Representative plots of CD57 expression on CD8^{bright} and CD8^{dim} T cells. B) Representative plots of Eomes expression on CD8^{bright} and CD8^{dim} T cells. C) CD3 and T-bet expression, comparing CD8^{bright} and CD8^{dim} T cells from HIV+ KS+ (gray open squares) and HIV+ KS^{neg} (black circles) participants.

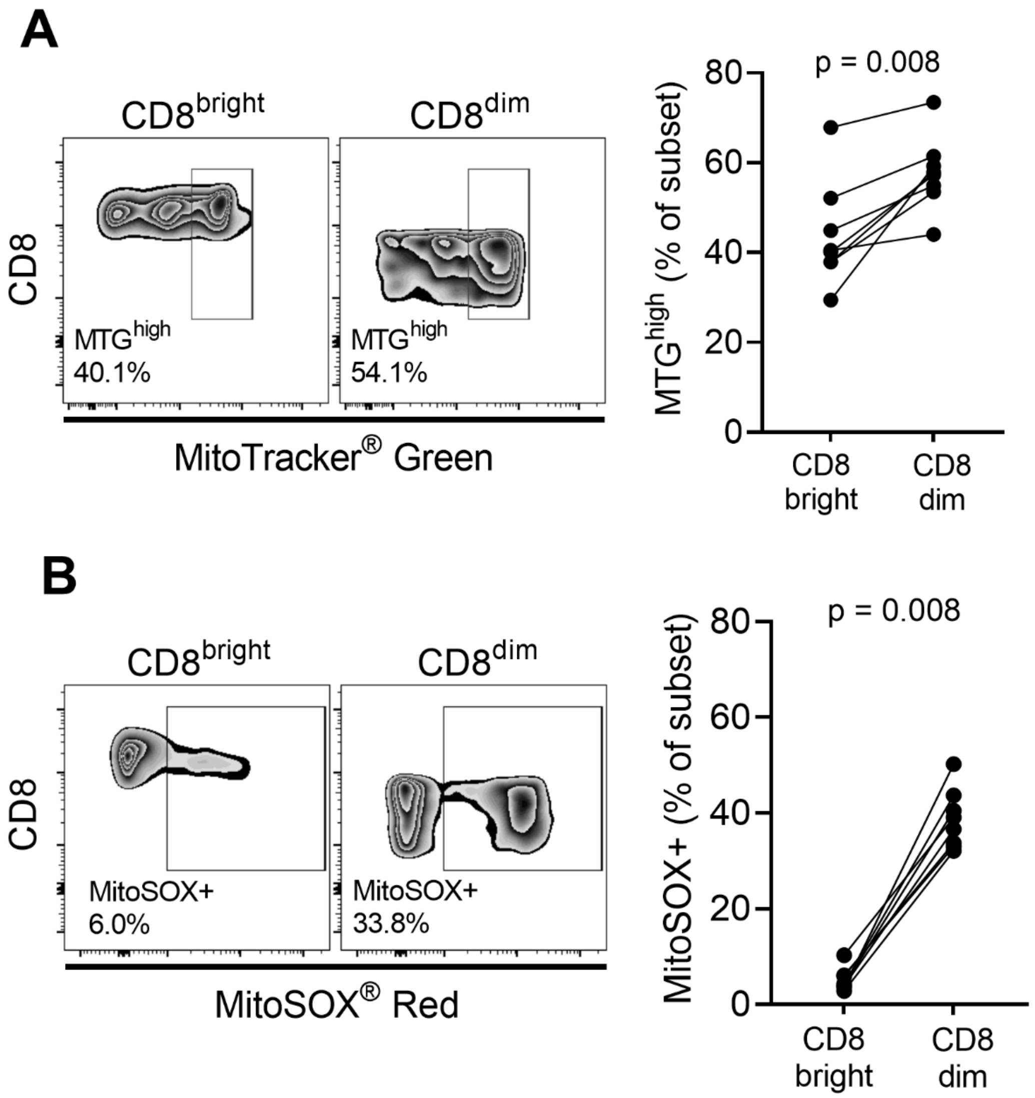

Supplementary Figure 4. A) Frequency of MitoTracker Green high cells, comparing CD8^{bright} and CD8^{dim} T cells from HIV+ KS^{neg} participants. B) Frequency of MitoSOX+ cells, comparing CD8^{bright} and CD8^{dim} T cells from HIV+ KS^{neg} participants.
